# Spontaneous Pneumothorax Revealing Pulmonary Alveolar Microlithiasis: A Case Report

**DOI:** 10.7759/cureus.104673

**Published:** 2026-03-04

**Authors:** Jamal Oujaber, Amine Harzimi Benjelloun, Soufiane Sassi, Mohamed Amine Azami, Hicham Janah

**Affiliations:** 1 Pulmonology Department, Avicenne Military Hospital, Marrakech, MAR; 2 Pathology and Laboratory Medicine Department, Avicenne Military Hospital, Marrakech, MAR

**Keywords:** calcospherites, miliary, pneumothorax, pulmonary alveolar microlithiasis, pulmonary calcifications

## Abstract

Pulmonary alveolar microlithiasis is a rare autosomal recessive lung disease characterized by intra-alveolar accumulation of calcium phosphate microliths. Diagnosis is often incidental on chest imaging, while histological examination remains the gold standard. We report the 15th case of pulmonary alveolar microlithiasis in Morocco, revealed by a complete spontaneous pneumothorax. A 61-year-old patient with a family history of chronic respiratory failure presented with acute chest pain and dyspnea. Chest radiography demonstrated diffuse micronodular opacities associated with a large pneumothorax. Initial management included pleural drainage; persistent air leakage required pleurodesis with simultaneous lung biopsies. Histopathological analysis confirmed the diagnosis by demonstrating intra-alveolar calcospherites. Two years after diagnosis, the patient reported exertional dyspnea. Pulmonary function testing demonstrated a restrictive pattern, and arterial blood gases revealed a PaO₂ of 73 mmHg, indicating mild hypoxemia. This case highlights the importance of histological confirmation in pulmonary alveolar microlithiasis, particularly in atypical presentations such as spontaneous pneumothorax.

## Introduction

Pulmonary alveolar microlithiasis (PAM) is a rare disorder of unknown etiology. In Morocco, only 14 cases have been reported in the literature, including eight female patients. The disease is characterized by the intra-alveolar accumulation of calcium phosphate microliths, occurring in the absence of any disturbance in calcium-phosphate metabolism. PAM remains asymptomatic for a prolonged period of time and is most often incidentally detected during routine chest imaging [1]. The most characteristic radiological feature is the presence of diffuse, fine calcified micronodules producing a typical “sandstorm” appearance, which is frequently sufficient to establish the diagnosis. Diagnostic confirmation relies on the identification of mutations in the SLC34A2 gene or the demonstration of microliths in bronchoalveolar lavage fluid or sputum. In some cases, transbronchial or surgical lung biopsy may be required [2-4]. We report a new case of pulmonary alveolar microlithiasis presenting with spontaneous pneumothorax as an unusual presentation, along with a review of the relevant literature.

## Case presentation

Our patient is a 61-year-old woman, a housewife, with no history of toxic habits, occupational exposure, or regular medication use. She had a sister and a brother who died from chronic respiratory disease; however, no medical records were available. She presented to the emergency department of our hospital with acute left-sided pleuritic chest pain, associated with dry cough and moderate dyspnea. The initial clinical examination revealed a conscious patient in good general condition with a heart rate of 100 beats per minute (bpm), a respiratory rate of 28/min, significant digital clubbing, and an oxygen saturation of 90% on air.

**Figure 1:**
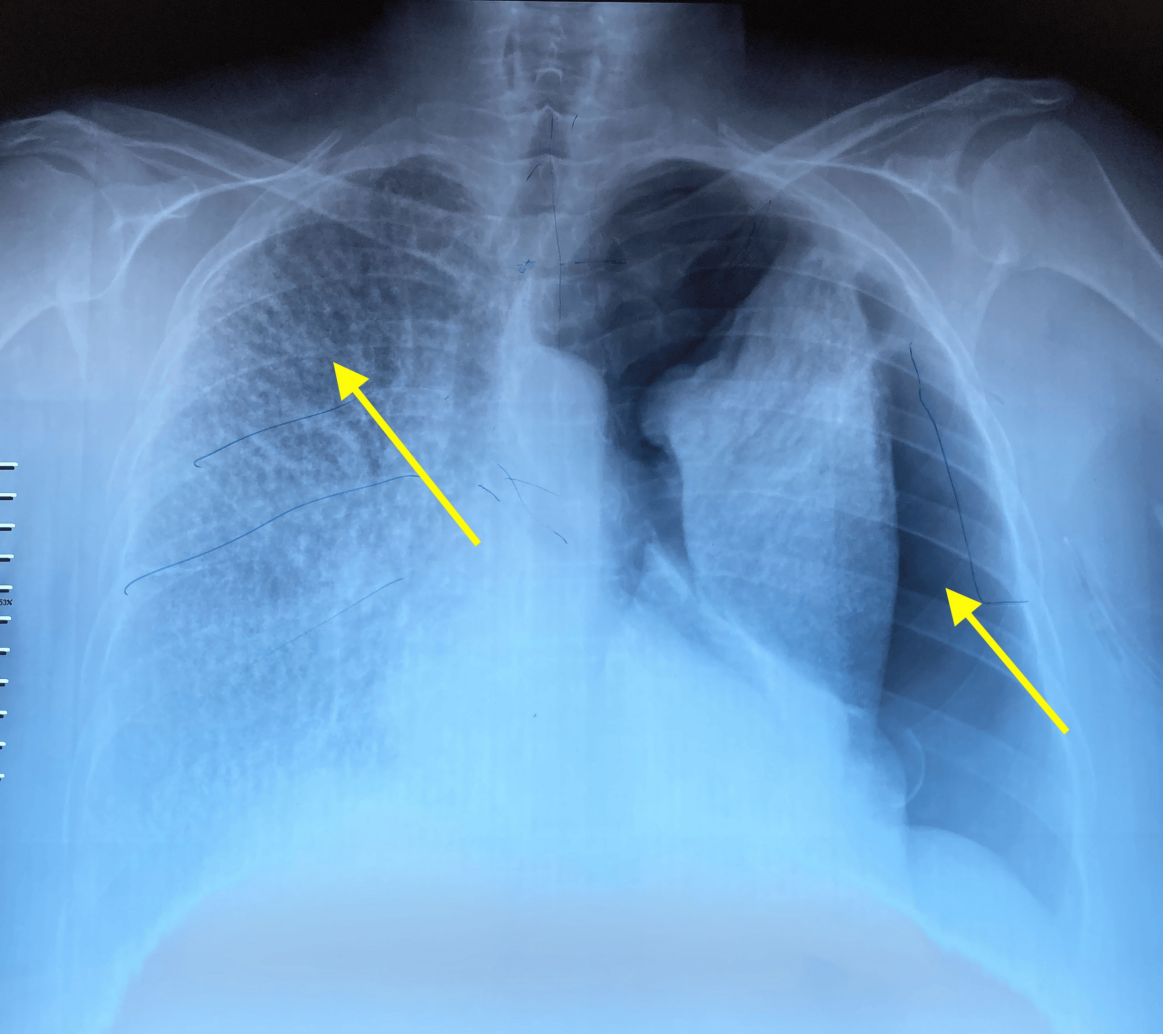
Initial chest radiograph of the patient The image is showing a complete left pneumothorax with a thin calcified miliary pattern on the right, sparing the apex (yellow arrows).

**Figure 2:**
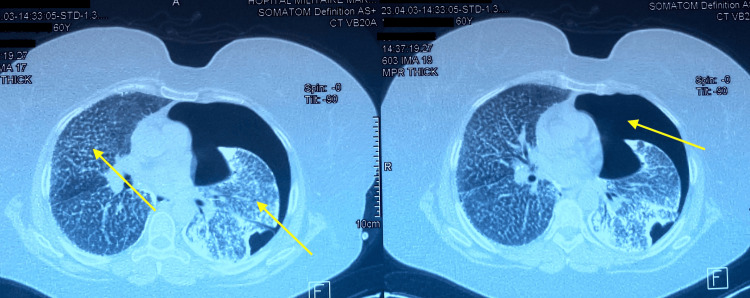
CT scan of the chest (axial view) The images show a left pneumothorax with calcified left pulmonary atelectasis and a right pulmonary micronodular interstitial syndrome.

On pleuropulmonary examination, decreased breath sounds were noted on the left side on auscultation, with the absence of vocal fremitus on palpation and tympanic percussion. On the right side, crackles were present. Chest radiography (Figure 1) shows on the left a peripheral hyperlucency without bronchovascular markings, with retraction of the lung parenchyma towards the hilum and a pleural-to-hilar distance greater than 3 cm, without severity criteria. On the right, a fine miliary pattern is observed with calcified micronodules sparing the apex. Thoracic computed tomography (CT) (Figure 2) revealed a large left-sided pneumothorax with calcified atelectasis of the left lung, as well as a right-sided micronodular interstitial lung pattern.

**Figure 3:**
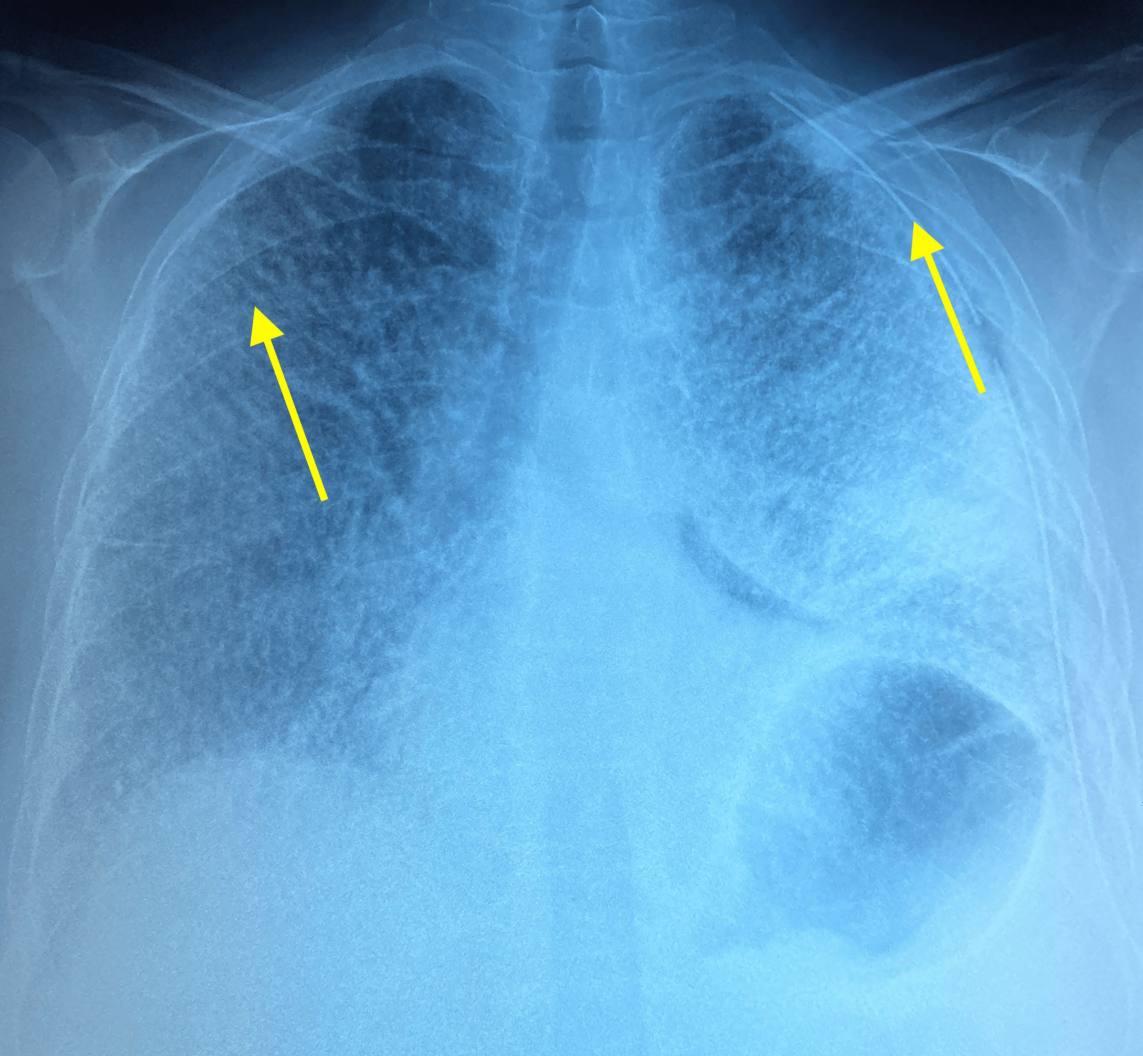
Chest radiograph obtained after pneumothorax drainage The image shows lung re-expansion against the chest wall with a drain in place (yellow arrow).

Laboratory investigations showed a C-reactive protein level of 6 mg/L. Serum calcium levels were within the normal range, as were electrolyte, hepatic, and renal function tests. The white blood cell count was 12.72 × 10⁹/L, and the hemoglobin level was 12.8 g/dL. Investigations for *Mycobacterium tuberculosis *were negative. Arterial blood gas analysis revealed a PaO₂ of 72 mmHg and a PaCO₂ of 40 mmHg. The patient underwent intercostal pleural drainage using a small-bore Jolly chest tube (<14 F), with an initially uncomplicated clinical course. A follow-up chest radiograph demonstrated lung re-expansion with the chest tube in place (Figure 3).

**Figure 4:**
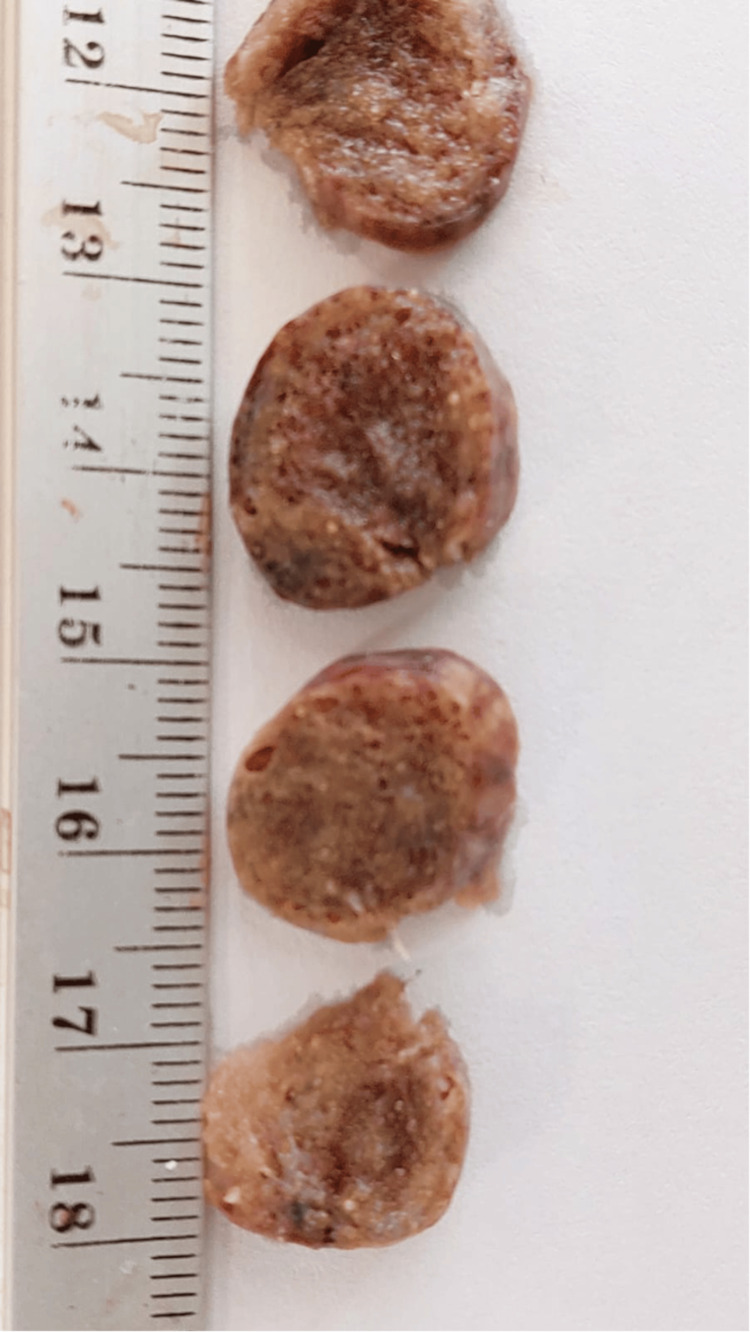
Lung biopsy samples (macroscopic appearance of lung fragments)

**Figure 5:**
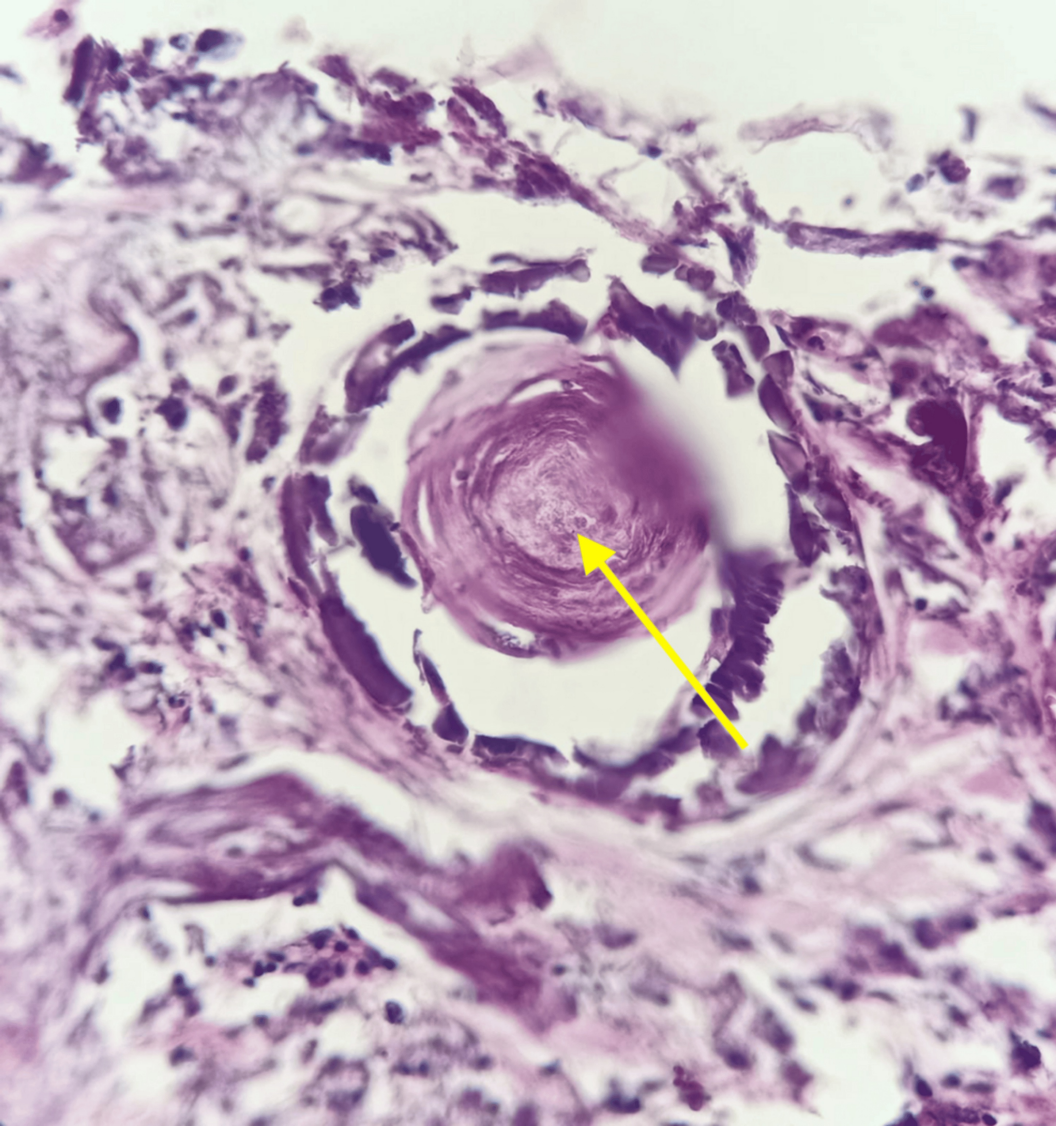
Pulmonary biopsy image The image shows the presence of calcospherites within the pulmonary alveolar spaces (yellow arrow).

The chest tube was connected to a continuous suction system; however, persistent air leakage beyond six days prompted talc pleurodesis, performed concomitantly with lung biopsies (Figure 4). Histopathological examination revealed numerous intra-alveolar calcospherites of variable size (Figure 5), confirming the diagnosis of pulmonary alveolar microlithiasis. Following successful pleurodesis, the chest tube was removed. During hospitalization, the patient received respiratory physiotherapy, which was continued for one week after discharge.

The patient has been regularly followed in the pulmonology outpatient clinic of our hospital for the past two years. She is up to date with both influenza and pneumococcal vaccinations. Her most recent spirometry demonstrated a restrictive ventilatory defect, with a vital capacity of 48% of the predicted value and an FEV₁ of 46% of predicted. Arterial blood gas analysis revealed a PaO₂ of 73 mmHg and a PaCO₂ of 38 mmHg. The patient has not yet been initiated on long-term oxygen therapy.

**Figure 6:**
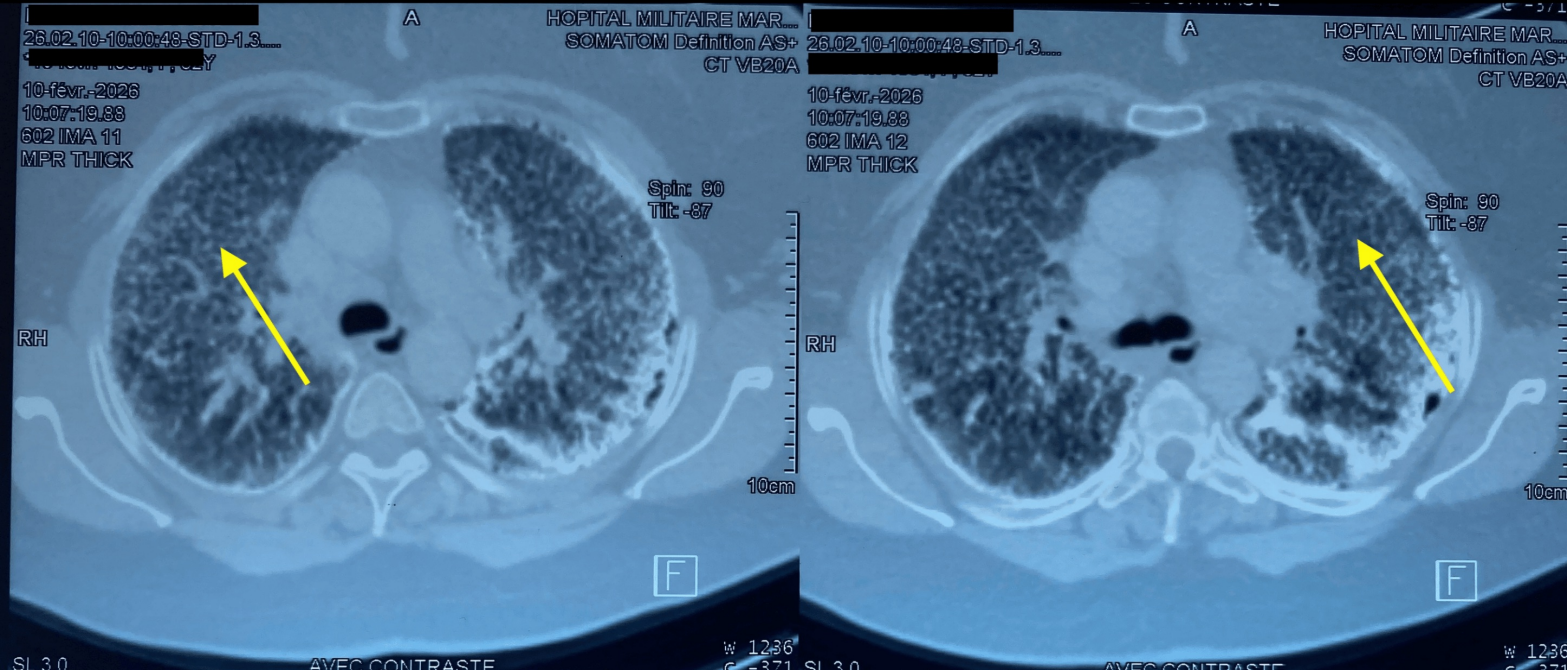
Chest CT scan (axial view) CT scan Images demonstrating bilateral diffuse micronodular interstitial involvement consistent with pulmonary alveolar microlithiasis (PAM) (yellow arrows).

Her latest follow-up chest CT scan (Figure 6) demonstrated diffuse bilateral intraparenchymal micronodules of submillimetric size, producing the classic “sandstorm” appearance. Subpleural pulmonary microcystic formations were also observed in the left upper lobe, findings consistent with pulmonary alveolar microlithiasis.

## Discussion

Pulmonary alveolar microlithiasis was first described in the 17th century [2]. In the early 20th century, additional cases were documented, providing more detailed clinicopathological and radiological characterizations of the disease. By the 1930s, the condition had been formally recognized and named pulmonary alveolar microlithiasis [2]. In total, just over 1100 cases have been published in the literature in Morocco [5], and 14 cases have been published, including eight female patients [1].

Pulmonary alveolar microlithiasis is a rare disease reported in all regions of the world, with a higher prevalence in Asia [2]. The mean age at diagnosis ranges between 20 and 50 years. There is no clear sex predilection. A familial form is identified in approximately 50% of cases, with an autosomal recessive mode of inheritance, and no environmental risk factor has been clearly identified [6]. More recently, mutations in the SLC34A2 gene, which are responsible for the disease, have been identified. This gene is highly expressed in both fetal and adult lungs and plays a key role in inorganic phosphate homeostasis, as it encodes a type II sodium-dependent phosphate cotransporter. Loss of function of this transporter leads to the formation and accumulation of calcium phosphate microliths within the alveolar spaces, as well as their deposition in the interlobular septa and pleural [6,7].

Macroscopically, the lungs lose their elasticity and may weigh up to 5 kg. The external surface appears irregular due to the protrusion of microliths into the visceral pleura. Bullae or blebs may be present, particularly in the anterior and apical regions of the lungs [8], as observed in our patient. Microliths are ovoid or round structures measuring between 0.01 and 2.8 mm, with a calcium-to-phosphate ratio of 2:1. Histologically, microliths stain positively with periodic acid-Schiff (PAS) and exhibit a characteristic concentric lamellar appearance surrounding a central nucleus [9].

Clinically, the disease often remains asymptomatic for a long period and is frequently discovered incidentally during routine radiological examinations. When present, symptoms may include dry cough, dyspnea, digital clubbing, or chest pain. Pulmonary function is initially preserved; however, as microliths accumulate, a restrictive ventilatory defect develops, leading to progressive dyspnea. In advanced stages, patients may develop respiratory failure, alveolar hypoventilation, and pulmonary hypertension [5].

On chest radiography, pulmonary alveolar microlithiasis typically presents as diffuse, bilateral micronodules of calcific density, producing the characteristic “sandstorm” appearance [10]. Chest computed tomography (CT) demonstrates calcified micronodules predominantly in the middle and basal lung zones, which may be confluent, and distributed along the bronchovascular bundles, interlobular septa, and peri- and centrilobular regions [11]. These typical radiographic and CT findings-diffuse, bilateral, basilar, sandstorm-like calcified micronodules-are often sufficient to establish the diagnosis, particularly when a family history is present, and may obviate the need for further investigations [2,5,8].

The differential diagnosis includes other causes of miliary lung disease, such as tuberculosis, sarcoidosis, pulmonary hemosiderosis, and pneumoconiosis. Clinicians should have in mind that some findings seen in pulmonary alveolar microlithiasis, such as nodular calcifications, can be found in other diseases like tuberculosis, metastatic osteosarcoma, amyloidosis, and silicoproteinosis. Besides that, dense consolidations can also be found in metastatic pulmonary calcification, talcosis, and amiodarone lung toxicity [12].

A definitive diagnosis can be confirmed by lung biopsy, which reveals microliths within the alveolar spaces associated with inflammatory changes. Microliths may also be identified in sputum samples and bronchoalveolar lavage (BAL) fluid. Transbronchial lung cryobiopsy is another diagnostic option [12]. Identification of a pathogenic mutation in the SLC34A2 gene provides molecular confirmation of the diagnosis [13]. The disease follows a slowly progressive course and may be complicated by recurrent respiratory infections, worsening dyspnea, pulmonary fibrosis, pulmonary hypertension, and ultimately chronic respiratory failure [14]. Therapeutic options remain limited. Treatments such as corticosteroids and disodium etidronate have been attempted without proven efficacy [4]. To date, lung transplantation remains the only intervention shown to improve survival in advanced disease [15].

## Conclusions

Pulmonary alveolar microlithiasis is a rare and often underdiagnosed condition that should be considered in patients presenting with calcified miliary opacities on chest imaging. The disease is typically characterized by minimal or nonspecific clinical manifestations despite extensive radiological involvement, with a slowly progressive course that may ultimately lead to chronic respiratory failure. Histopathological confirmation remains essential for definitive diagnosis, particularly in atypical presentations such as spontaneous pneumothorax. Currently, no effective medical therapy has been established, and lung transplantation remains the only curative treatment option in advanced stages.
